# The Cortisol Effect on the NO/cGMP Pathway

**DOI:** 10.3390/ijms26041421

**Published:** 2025-02-08

**Authors:** Maria Grazia Signorello, Giuliana Leoncini

**Affiliations:** Biochemistry Laboratory, Department of Pharmacy, University of Genoa, 16132 Genova, Italy; giuliana.leoncini@libero.it

**Keywords:** cortisol, cGMP, human platelets, nitric oxide, nitric oxide synthase, reactive nitrogen species

## Abstract

Previously, it has been shown that cortisol induces oxidative stress in human platelets, stimulating reactive oxygen species production, superoxide anion formation, lipid peroxidation, and depleting antioxidant defenses. In this study, the cortisol effect on platelet function has been described. Results demonstrate that cortisol stimulates platelet activation and aggregation, leading to CD62P surface exposure and intracellular calcium elevation. Cortisol potentiates its aggregating effect, reducing the level of the powerful anti-aggregating agent nitric oxide (NO). Likewise, cortisol reduces cGMP levels. Moreover, specific inhibitors of the Src/Syk/PI3K/AKT pathways reverse the inhibiting effect of cortisol, partially restoring NO and cGMP levels. Unexpectedly, cortisol stimulates endothelial nitric oxide synthase (eNOS) activity, measured in platelet lysates prepared by whole cells treated with the hormone. The phosphorylation of the Ser1177 eNOS activating-residue is increased by cortisol. The Src/Syk/PI3K/AKT pathways appear to be involved in the phosphorylation of this residue. Moreover, cortisol induces the formation of nitrotyrosine, that can be considered a biomarker for reactive nitrogen species, including peroxynitrite. In conclusion, through these mechanisms, cortisol potentiates its capacity to induce oxidative stress in human platelets.

## 1. Introduction

Cortisol is a steroid hormone that is produced and released by the adrenal cortex of vertebrates. As a hormone, it modulates cellular responses to various stimuli, and since the main one that involves cortisol is stress, it is commonly known as the stress hormone [1]. High cortisol levels can lead to cardiovascular diseases such as hypertension, vascular atherosclerosis, cardiac remodeling, and thrombosis [2,3]. In fact, patients with Cushing’s syndrome, a pathological condition characterized by persistently high cortisol levels, have been observed to present hypercoagulability [4], venous thromboembolism [5], and an increased risk of cardiovascular events [6]. Enhanced oxidative stress and platelet activation in patients affected by Cushing’s syndrome have been described [7]. Moreover, plasma levels of factor VIII of the coagulation cascade and the von Willebrand factor are markedly increased in these patients [8]. Concerning the effects of cortisol on platelet function, opposing results have been published. Two reports have shown that cortisol may produce platelet hyperactivation [9] and that hypercortisolism is associated to platelet hyperreactivity [8], whereas other studies have reported an inhibitory effect of exogenous glucocorticoids on platelet aggregation [10,11,12]. In addition, negative correlations have been demonstrated between plasma cortisol concentration and platelet reactivity in response to arachidonic acid and ADP in older adults [13]. Nitric oxide (NO) plays a crucial role in numerous physiological processes, including the regulation of vascular tone and the inhibition of platelet adhesion, aggregation, and secretion [14,15]. The biochemical mechanisms of NO can be summarized by three key reactions: activation of guanylate cyclase, which mediates signal transduction; degradation through interaction with oxyhemoglobin; and conversion into reactive nitrogen species, specifically peroxynitrite, through a reaction with superoxide anions. In blood vessels, NO is predominantly synthesized by endothelial nitric oxide synthase (eNOS, type III) and its production is stimulated by factors such as insulin, shear stress, and other agents that elevate intracellular calcium levels. Platelets may also contribute to NO production via two isoforms of nitric oxide synthase: endothelial NOS (eNOS) and inducible NOS (iNOS). However, the expression and enzymatic activity of NOS in platelets remain poorly understood [16,17]. On the other hand, eNOS protein has been identified in platelets by Western blotting technique [18]. Platelet eNOS is considered a calcium-independent enzyme, and the phosphorylation/dephosphorylation of the Ser1177 and/or Thr495 residues plays an important role in the regulation of its activity. The phosphorylation of Ser1177 residue activates eNOS, while the phosphorylation of Thr495 residue inhibits the activity of the enzyme [19]. The NO effects, including vascular tone and platelet function, are mediated by cGMP that is produced by the NO activated soluble guanylyl cyclase [15,20]. A defect in NO/cGMP signaling promotes vasoconstriction, inflammation, and thrombosis associated with major cardiovascular risk [21].

To further contribute to understanding how cortisol can activate platelets, we have measured aggregation, CD62 exposure, calcium levels, NO, and cGMP levels. In addition, we have checked eNOS activity and its phosphorylation status upon cortisol treatment and the role of the Src/Syk, PI3K/AKT pathways in these mechanisms. Finally, nitrotyrosine, a biomarker of reactive nitrogen species formation, including peroxynitrite, in cortisol treated-platelets, was measured.

## 2. Results

### 2.1. Effect of Cortisol on Platelet Aggregation, CD62P Exposure, and Calcium Elevation

**Figure 1:**
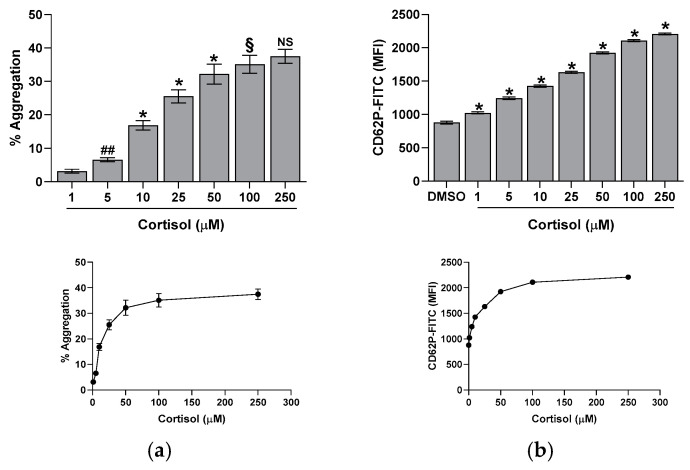
Effect of cortisol on platelet aggregation (panel (**a**)) and CD62P exposure (panel (**b**)). Assays were performed as described in the Methods Section. Reported data are the mean ± SD of at least five determinations carried out in duplicate. One-way ANOVA and Tukey’s post hoc test relate to panels (**a**,**b**): * *p* < 0.0001, ## *p* < 0.005, § *p* < 0.01, and NS—not significant.

We studied the effect of varying cortisol concentrations on platelet aggregation, CD62P exposure, and intracellular calcium. We found that cortisol stimulates platelet aggregation in a dose-dependent manner, with a peak response at 50 µM (Figure 1a).

**Figure 2:**
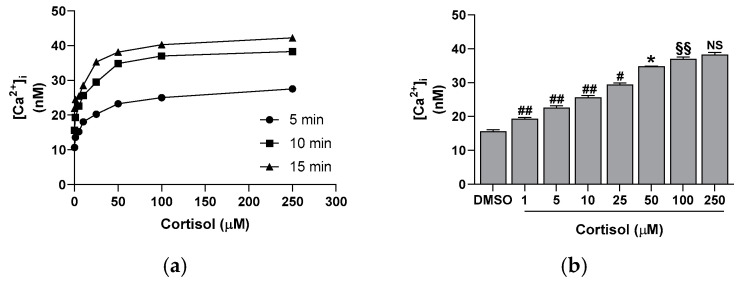
Effect of cortisol on [Ca^2+^]_i_ levels. [Ca^2+^]_i_ was determined in FURA 2-loaded platelets, as detailed in the Methods Section. In panel (**a**), the time course of calcium rise in response to the reported cortisol concentrations is shown. In panel (**b**) is detailed the effect of 10 min incubation with cortisol. Reported data are the mean ± SD of at least five determinations carried out in duplicate. One-way ANOVA and Tukey’s post hoc test relate to panels (**a**,**b**): * *p* < 0.0001, # *p* < 0.001, ## *p* < 0.005, §§ *p* < 0.05, and NS—not significant.

Similarly, cortisol stimulates platelet activation, leading to the CD62P surface exposure. The hormone effect is dose-dependent, peaking at 50 µM (Figure 1b) and showing a good correlation with platelet aggregation (y = 33.097x + 900.85; r^2^ = 0.9759). Moreover, cortisol triggers the release of intracellular calcium in a dose- and time-dependent manner, reaching a maximum at 50 µM and at 10 min of incubation (Figure 2). The calcium rise is also closely related to platelet aggregation (y = 0.5617x + 16.764; r^2^ = 0.9756) and CD62P exposure (y = 0.0169x + 1.5203; r^2^ = 0.9963).

### 2.2. Effect of Cortisol on NO and cGMP Basal Levels

**Figure 3:**
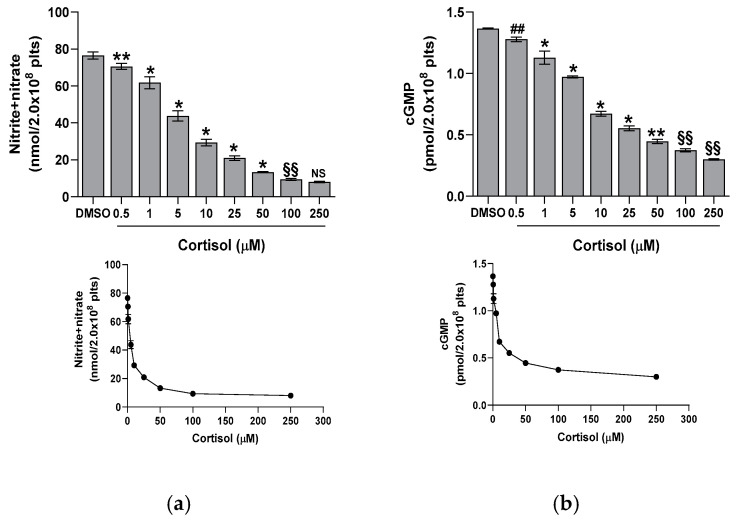
Effect of cortisol on nitrite + nitrate (panel (**a**)) and cGMP (panel (**b**)) basal levels. Nitrite + nitrate and cGMP were determined by commercial kits, as described in the Methods Section. Reported data are the mean ± SD of at least five determinations carried out in duplicate. One-way ANOVA and Tukey’s post hoc test relate to panels (**a**,**b**): * *p* < 0.0001, ** *p* < 0.0005, ## *p* < 0.005, §§ *p* < 0.05, and NS—not significant.

**Figure 4:**
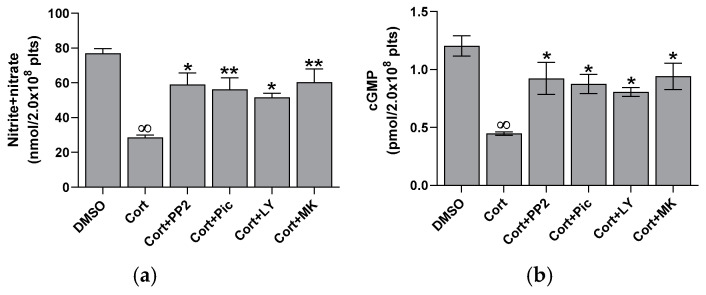
Effect of inhibitors on nitrite + nitrate (panel (**a**)) and cGMP (panel (**b**)) formation in cortisol-treated platelets. Washed platelets were preincubated with DMSO or 10 µM PP2, 30 µM piceatannol (Pic), 20 µM LY294002 (LY), 20 µM MK2206 (MK), and then challenged with 50 µM cortisol (Cort). Nitrite + nitrate and cGMP were determined by commercial kits, as detailed in the Methods Section. Reported data are the mean ± SD of at least five determinations carried out in duplicate. Multiple unpaired *t*-tests relate to panels (**a**,**b**): ∞ *p* < 0.0001 vs. DMSO; * *p* > 0.0001 and ** *p* < 0.0005 vs. Cort.

Cortisol significantly reduces platelet basal nitrite + nitrate levels at all tested concentrations, reaching the maximal effect at 50 µM (Figure 3a). Nitrite and nitrate are the stable end products of NO in vivo; therefore, their sum is commonly used as an indicator of NO levels in vivo [22]. Since NO can activate soluble guanylyl cyclase to produce cGMP, we further examined the impact of cortisol on this pathway. A dose-dependent decrease in cGMP levels was observed (Figure 3b), indicating a direct link between NO and cGMP. A strong correlation between NO and cGMP levels was confirmed by the regression analysis (y = 0.0151x + 0.2316; r^2^ = 0.9892). To closely examine the signaling pathways involved, we evaluated the effect of several specific inhibitors of transduction steps on nitrite + nitrate and cGMP levels in platelets treated with cortisol. As shown in Figure 4, PP2, piceatannol, LY294002, and MK2206, inhibitors of Src, Syk, PI3K, and AKT, respectively, reversed the inhibiting effect of cortisol, partially restoring nitrite + nitrate (panel a) and cGMP (panel b) levels. 

### 2.3. Effect of Cortisol on eNOS Activity

**Figure 5:**
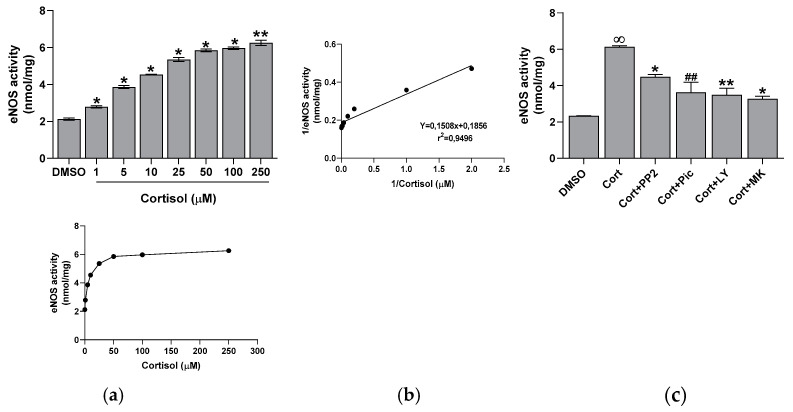
Effect of cortisol on eNOS activity. Whole cell lysates were preincubated with DMSO, 10 µM PP2, 30 µM piceatannol (Pic), 20 µM LY294002 (LY), or 20 µM MK2206 (MK), and then treated with cortisol, as indicated in panels (**a**,**b**), or challenged with 50 µM cortisol, as indicated in panel (**c**). eNOS activity was then evaluated as reported in the Methods Section. One-way ANOVA and Tukey’s post hoc test relate to panel (**a**): * *p* < 0.0001, ** *p* < 0.0005; multiple unpaired *t*-tests relate to panel (**c**): ∞ *p* < 0.0001 vs. DMSO; * *p* < 0.0001, ** *p* < 0.0005, and ## *p* > 0.005 vs. Cort.

We measured eNOS activity in platelet lysates derived from whole cells incubated with increasing concentrations of cortisol. Unexpectedly, we found that cortisol dose-dependently stimulated eNOS activity (Figure 5a), with the effect being significant at all tested concentrations. The inducing effect of cortisol on eNOS activity was inversely correlated with the decrease in NO levels (y = −0.0603x + 6.5751; r^2^ = 0.9915). Additionally, kinetic parameters for eNOS activity towards cortisol were determined, yielding a Km of 1.23 µM and a Vmax of 41.31 nmol/mg (Figure 5b). To confirm the involvement of the Src, Syk/PI3K, and AKT pathways, we tested the effect of specific inhibitors of these enzymes. All inhibitors led to a significant reduction in cortisol-induced eNOS activity (Figure 5c).

**Figure 6:**
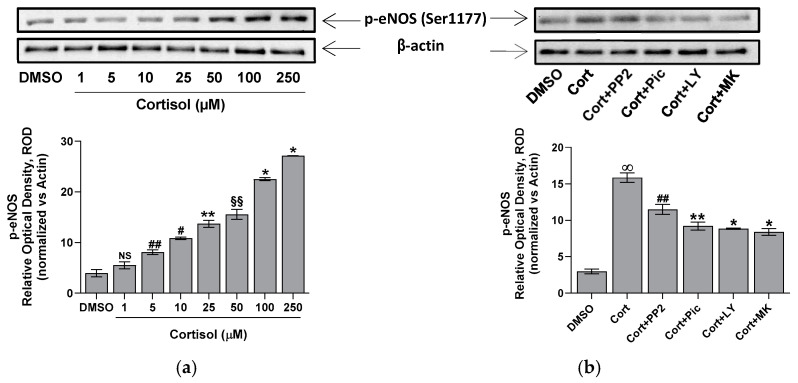
The cortisol effect on Ser1177 eNOS phosphorylation. Washed platelets were preincubated with DMSO, 10 µM PP2, 30 µM piceatannol (Pic), 20 µM LY294002 (LY), or 20 µM MK2206 (MK), and then treated with cortisol, as indicated in panel (**a**), or challenged with 50 µM cortisol, as indicated in panel (**b**). Bar charts show the densitometric analysis of signals expressed as relative optical density (ROD), normalized against the actin signal, used as housekeeping protein. Reported bars are the mean ± SD of at least three experiments. One-way ANOVA and Tukey’s post hoc test relate to panel (**a**): * *p* < 0.0001, ** *p* < 0.0005, # *p* < 0.001, ## *p* < 0.005, §§ *p* < 0.05, and NS—not significant. Multiple unpaired *t*-tests relate to panel (**b**): ∞ *p* < 0.0001 vs. DMSO; * *p* < 0.0001, ** *p* < 0.0005, and ## *p* > 0.005 vs. Cort.

The phosphorylation of eNOS Ser1177 activates the enzyme [19]. Thus, the phosphorylation status of eNOS at Ser1177 in platelets treated with cortisol has been evaluated. Data in Figure 6a indicate that cortisol positively modulates the phosphorylation of the Ser1177 residue. This phosphorylation appears to be dependent on the Src/Syk/PI3K/AKT pathways, as pretreatment of platelets with PP2, Piceatannol, LY294002, or MK2206 significantly reduces cortisol-induced phosphorylation of the eNOS Ser1177 residue (Figure 6b).

### 2.4. Effect of Cortisol on Nitrotyrosine Formation

**Figure 7:**
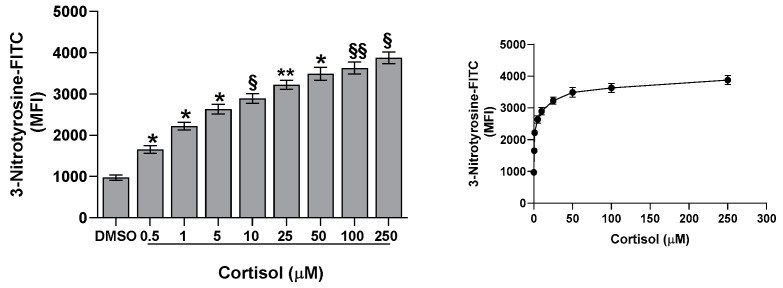
Effect of cortisol on nitrotyrosine. Nitrotyrosine was determined by flow cytometry, as reported in the Methods Section. One-way ANOVA and Tukey’s post hoc test: * *p* < 0.0001, ** *p* < 0.0005, § *p* < 0.01, and §§ *p* < 0.05.

Previously, we have shown that cortisol stimulates ROS and superoxide anion formation [23]. In the current study, we describe a dose-dependent activation of eNOS, but we do not observe a corresponding increase in NO and cGMP levels. Thus, we hypothesize that the decreased NO availability could be explained by the formation of reactive nitrogen species, including peroxynitrite. These species, measured through nitrotyrosine formation, are increased in platelets treated with cortisol, being the effect dose-dependent and peaking at 50 µM (Figure 7). Interestingly, nitrotyrosine levels show a positive correlation with eNOS activity (y = 628.9x + 39.023; r^2^ = 0.9393) and an inverse correlation with NO formation (y = −38.269x + 4186.3; r^2^ = 0.9496), suggesting that the increased eNOS activity promotes reactive nitrogen species formation, which may, in turn, contribute to the observed decrease in NO levels.

## 3. Discussion

It is known that glucocorticoids exert a noteworthy impact in hemostasis [24,25,26]. Many studies suggest that these hormones may influence hemostasis and fibrinolysis, potentially directing to thromboembolic events [4,5,6,8,27]. However, even if glucocorticoid receptors leading to nongenomic responses have been recently demonstrated in platelet cytoplasm [9,12], the effect of hypercortisolemia on platelet function is less clear. Some studies report an inhibitory effect of glucocorticoids on platelet function and in particular affecting platelet aggregation in animals [10,11,12,28]. Other studies, on the contrary, report platelet hyperreactivity [9], spontaneous platelet aggregation, and hyperresponsiveness to ristocetin due to von Willebrand multimers overexpression observed in hypercortisolism [8]. Previously, we have shown that cortisol induces oxidative stress stimulating ROS production, superoxide formation, and lipid peroxidation [23] in human platelets. The involvement of Src, Syk, PI3K, AKT, NADPH oxidase 1 enzymes, and complexes I and II of oxidative phosphorylation was demonstrated [23]. As a support, Karamouzis et al. [7] have shown enhanced oxidative stress and platelet activation in patients affected by Cushing’s syndrome. In this study, we demonstrate that cortisol, probably involving its specific glucocorticoid receptor [9,12], acts as a true agonist, stimulating platelet activation and aggregation, and calcium elevation in a dose-dependent manner, being the effect significant at all tested concentrations (Figure 1 and Figure 2). Interestingly, the lowest cortisol concentration used in our experiments is close to the physiological serum cortisol levels usually reported in healthy subjects, which range from 0.4 to 0.6 µM [29,30], but that markedly increase two- or three-fold in Cushing’s syndrome patients [29]. Since cortisol behaves as a platelet agonist, we assessed its effect on the NO/cGMP pathway. NO inhibits platelets by inhibiting platelet adhesion, activation, and aggregation, and by disaggregating platelets that were previously aggregated [31]. Thus, as expected, a reduction in NO availability was observed, as shown in Figure 3a. In strict correlation, cGMP also decreases (Figure 3b). Subsequently, we have tested eNOS activity in platelet lysates obtained from whole cells treated with cortisol. Unexpectedly, we have found that cortisol stimulates eNOS activity (Figure 5). The mechanisms underlying NOS activation in platelets, as well as in other cells, involve an increase in intracellular calcium levels, phosphorylation of the Ser1177 residue, and/or dephosphorylation of the Thr495 residue [19]. As reported in Figure 6a, cortisol dose-dependently increases the phosphorylation of the activating residue Ser1177. This phosphorylation is significantly inhibited by PP2, piceatannol, LY294002, and MK2206, suggesting that the Src and Syk/PI3K/AKT pathways may play a role in mediating the hormone’s effect (Figure 6b). Cortisol does not modify the phosphorylation level of Thr495 residue (we carried out the experiments and did not observe any changes in Thr495 eNOS phosphorylation induced by cortisol). The decrease in NO basal levels and the eNOS phosphorylation/activation produced by cortisol are inversely correlated. Likely, we suggest that NO generated through eNOS activation can be very quickly transformed to reactive nitrogen species such as the oxidant peroxynitrite by the reaction with superoxide anion [32]. On the other hand, the reduced levels of the essential cofactor tetrahydrobiopterin cause the uncoupling of eNOS, leading to the production of superoxide anion instead of NO [33]. In addition, peroxynitrite can play a pivotal role in eNOS uncoupling [34,35]. The oxidation of tetrahydrobiopterin to the inactive pterin by peroxynitrite induces the dissociation of the cofactor from eNOS, leading to superoxide anion production [36]. Previously [23], we have demonstrated that superoxide anion is increased in cortisol-treated platelets. In this study, we have observed an increase in nitrotyrosine levels, usually considered as a biomarker of reactive nitrogen species production, including peroxynitrite (Figure 7) [37]. Likely, an increased formation of peroxynitrite stimulated by cortisol could be suggested. Peroxynitrite modifies tyrosine in proteins to nitrotyrosine, and the nitration of structural proteins, including neurofilaments and actin, can disrupt filament assembly with pathological consequences, as observed in human atherosclerosis [38], chronic coronary syndrome [39], and some neuronal diseases [40].

## 4. Materials and Methods

### 4.1. Materials

Apyrase, L-arginine, bovine serum albumin, BH_4_, cortisol, digitonin, dithiothreitol, FAD, FMN, leupeptin, NADPH, paraformaldehyde, PGE_1_, phenyl methyl sulfonyl fluoride (PMSF), piceatannol, PP2 analogue (PP2), protease inhibitor cocktail (Cat. N° P8340), Triton X-100, Tween-20, and all chemicals were purchased from Sigma-Aldrich, Darmstadt, Germany. FURA 2/AM and LY294002 were purchased from Merck Biosciences, Germany. MK2206 was purchased from Selleck Chemicals, Houston, TX, USA. Inhibitors were diluted from a stock DMSO solution immediately before each experiment. The nitric oxide detection kit was purchased from OzBiosciences, Marseille, France. The cGMP EIA kit was purchased from Cayman Chemical, Ann Arbor, MI, USA. Anti-actin, anti-CD62P-FITC, anti-3-nitrotyrosine-FITC, anti-phospho-eNOS Ser1177 and horseradish peroxidase-conjugated secondary antibodies were purchased from Santa Cruz Biotechnology, Dallas, TX, USA. Nitrocellulose membranes (pore size 0.45 µm) and 4–20% precast polyacrylamide gels were purchased from Bio-Rad Laboratories, Hercules, CA, USA. The ECL™ system was purchased from GE Healthcare, Cincinnati, OH, USA.

### 4.2. Blood Collection and Preparative Procedure

Fresh venous blood was collected from healthy volunteers at the Centro Trasfusionale, Ospedale San Martino in Genoa. The blood was drawn into an anticoagulant solution containing 130 mM aqueous trisodium citrate (9:1 ratio). Prior to collection, donors confirmed they had not taken any medications known to affect platelet function for at least two weeks, and they provided informed consent. Platelets were isolated by centrifuging whole blood at 100× *g* for 25 min to obtain platelet-rich plasma (PRP). Apyrase (4 mU/mL) and PGE1 (4 µM) were then added to the PRP before a second centrifugation at 1100× *g* for 15 min. The resulting pellet was washed with a pH 5.2 ACD solution (containing 75 mM trisodium citrate, 42 mM citric acid, and 136 mM glucose), followed by another centrifugation at 1100× *g* for 15 min. Finally, the pellet was resuspended in calcium-free HEPES buffer containing 10 mM HEPES, 145 mM NaCl, 5 mM KCl, 1 mM MgSO4, and 10 mM glucose (pH 7.4).

### 4.3. Aggregation Studies

Platelet aggregation was measured using a Bio-Data Aggregometer (Bio-Data Corporation, Horsham, PA, USA) following Born’s method [41]. Aggregation was quantified by monitoring six-minute light transmission. Washed platelets (3.0 × 10^8^ platelets/mL) were preincubated with DMSO at 37 °C before cortisol was added.

### 4.4. Flow Cytometric Analysis of CD62P

Washed platelets (1.0 × 10^9^ platelets/mL) were preincubated with DMSO at 37 °C, followed by stimulation with cortisol at 37 °C for 15 min. After incubation, appropriate aliquots of the samples were fixed in 2% paraformaldehyde in PBS for 30 min at 4 °C. Subsequently, anti-CD62P-FITC antibody was added, and each sample was analyzed using a Merck Millipore Bioscience Guava EasyCyte flow cytometer (Merck Millipore Biosciences, Darmastadt, Germany).

### 4.5. Intracellular [Ca^2+^] Measurement

Washed platelets (3.0 × 10^8^ platelets/mL) were incubated with 1 µg/mL FURA-2/AM for 45 min at 37 °C. Prior to centrifugation at 1100× *g* for 15 min, 2 µM PGE_1_ and 1 mM EGTA were added. The resulting pellet was resuspended at 2.0 × 10^8^ platelets/mL in calcium-free HEPES buffer and preincubated with DMSO at 37 °C, after which cortisol was added. Fluorescence from the FURA-2/AM-loaded platelets was measured at 37 °C under unstirred conditions using a Perkin-Elmer fluorescence spectrometer (model LS50B, Perkin Elmer, Shelton, CT, USA). Excitation wavelengths were set at 340 nm and 380 nm, with emission at 509 nm. The fluorescence intensity of fully saturated FURA-2/AM (Fmax) was determined by lysing the cells with 50 µM digitonin in the presence of 2 mM Ca^2+^, while Fmin was established by exposing the lysed platelets to 1 M EGTA. Autofluorescence was measured after fully quenching the signal with 5 mM Mn^2+^. Data were analyzed using software integrated with the spectrometer, which converted the fluorescence readings into cytosolic Ca^2+^ concentrations, with a Kd value for FURA-2/AM and Ca^2+^ of 135 nM.

### 4.6. Nitrite + Nitrate Measurement

Washed platelets (1.0 × 10^9^ platelets/mL), preincubated at 37 °C with DMSO or specific inhibitors, were stimulated with cortisol for 15 min in the presence of 100 μM L-arginine. The incubation was terminated by placing the samples on ice. Nitrite + nitrate levels were measured using a commercial kit, following the manufacturer’s instructions for the Griess reagent. Absorbance at 540 nm was recorded in a 96-well plate using a Bio-Rad Laboratories iMark^®^ microplate reader (Bio Rad Laboratories, Hercules, CA, USA).

### 4.7. cGMP Determination

Washed platelets (1.0 × 10^9^ platelets/mL) were prewarmed at 37 °C with DMSO or inhibitors and then incubated for 15 min in the presence of 100 µM L-arginine and cortisol. The reaction was terminated by adding cold 2 M perchloric acid. The precipitated proteins were removed by centrifuging the samples at 12,000× *g* for 2 min at 4 °C. The supernatants were neutralized with 2 M NaOH and immediately analyzed using a cGMP-specific EIA kit, based on competitive ELISA, following the manufacturer’s protocol.

### 4.8. eNOS Assay in a Cell-Free System

Washed platelets (2.0 × 10^9^ platelets/mL) were sonicated twice for 15 s on ice in the presence of 1 mM PMSF, 10 µg/mL leupeptin, 100 µM dithiothreitol, and a 1/100 dilution of protease inhibitor cocktail. The samples were then centrifuged at 600× *g* for 20 min. Appropriate aliquots of the supernatants were mixed with 100 µM NADPH, 10 µM FAD, 10 µM FMN, 0.1 µM BH_4_, 1 mM CaCl_2_, and 100 µM L-arginine, and incubated for 15 min at 37 °C with cortisol. The reaction was stopped by placing the samples on ice, and nitrite + nitrate levels were determined using a commercial kit, as previously described.

### 4.9. Immunoblotting Analysis

Washed platelets (1.0 × 10^9^ platelets/mL), preincubated with DMSO or inhibitors, were stimulated with cortisol and 100 μM L-arginine for 15 min at 37 °C. The reaction was terminated by adding 2× Laemmli-SDS reducing sample buffer, and the samples were heated at 100 °C for 5 min. Denaturing electrophoresis (SDS-PAGE) was performed on 4–20% gradient gels, loading 30 μg of protein per sample, followed by transfer to nitrocellulose membranes. The membranes were blocked with 5% BSA in TBST (Tris-buffered saline, pH 7.6, containing 10 mM Tris, 150 mM NaCl, and 0.1% Tween 20) at 25 °C for 60 min. They were then incubated overnight at 4 °C with anti-phospho-Ser1177 eNOS and anti-actin antibodies (both at a 1:2000 dilution in TBST). After extensive washing, the blots were incubated for 60 min at room temperature with horseradish peroxidase-conjugated secondary antibody (1:1000 in TBST). Following additional washes, the blots were developed using the ECL™ system. Band density, reported as fold-change relative to the control and normalized to β-actin, was quantified using the Bio-Rad Chemi-Doc software package (Quantity One 4.6.6 version).

### 4.10. Nitrotyrosine Assay

Nitrotyrosine is commonly used as a biomarker of reactive nitrogen species formation, including peroxynitrite [37]. Briefly, washed platelets (1.0 × 10^9^ platelets/mL) were preincubated at 37 °C with DMSO and subsequently stimulated with cortisol and 100 μM L-arginine for 15 min. After incubation, appropriate aliquots of the samples were fixed in 2% paraformaldehyde in PBS for 30 min at 4 °C and permeabilized with 1% Triton X-100 in PBS. Anti-3-nitrotyrosine-FITC antibody was then added, and each sample was analyzed using a Merck Millipore Bioscience Guava EasyCyte flow cytometer.

### 4.11. Statistical Analysis

Data are presented as the mean ± SD of at least five independent experiments, each conducted in duplicate. Statistical comparisons between two groups were performed using the multiple unpaired *t*-test. For comparisons involving multiple groups, one-way ANOVA followed by Tukey’s post hoc test was applied. A *p*-value of less than 0.05 was considered statistically significant.

## 5. Conclusions

In conclusion, cortisol behaves as a platelet agonist by stimulating activation, aggregation, and calcium elevation, and potentiates its aggregating effect through the reduction in NO availability. Reactive nitrogen species, and specifically peroxynitrite produced by the interaction of NO with superoxide anions, also generated through eNOS uncoupling, may contribute to this reduction. Thus, through these mechanisms, cortisol amplifies its capacity to induce oxidative stress in human platelets.

## Data Availability

The data presented in this study are available on request from the corresponding author upon reasonable request.
